# Prevalence of Intimate Partner Violence in Thailand

**DOI:** 10.1007/s10896-018-9960-9

**Published:** 2018-04-07

**Authors:** Montakarn Chuemchit, Suttharuethai Chernkwanma, Rewat Rugkua, Laddawan Daengthern, Pajaree Abdullakasim, Saskia E. Wieringa

**Affiliations:** 1 College of Public Health Sciences, Chulalongkorn Univerisity, Institute Building 2, Phayathai Road, Patumwan, Bangkok, 10330 Thailand; 2 The Royal Thai Ministry of Public Health, Nonthaburi, Thailand; 3 Faculty of Nursing, Naresuan University, Phitsanulok, Thailand; 4 Faculty of Public Health, Burapha University, Chonburi, Thailand; 5 The Amsterdam Institute for Social Science Research, University of Amsterdam, Amsterdam, The Netherlands

**Keywords:** Intimate partner violence, Domestic violence, Thailand, National prevalence

## Abstract

There is no recent national data on the prevalence of intimate partner violence in Thailand. This study proposed to examine the prevalence of intimate partner violence in 4 regions of Thailand by using a standardized questionnaire from the WHO multi country study on women’s health and domestic violence. Two thousand four hundred and sixty-two married or cohabiting women aged 20–59 years were interviewed about their experiences of psychologically, physically, sexually violent, and/or controlling behaviors by their male partners*.* The study found that 15% of respondents had experienced psychological, physical, and/or sexual violence in their life time which suggests that 1 in 6 of Thai women have faced intimate partner violence. Of the 15% of women who reported intimate partner violence within the past 12 months, psychological violence was the most common (60–68%), followed by sexual violence (62–63%) and physical violence (52–65%). In addition, the percentage of women who faced various forms of controlling behaviors varied from 4.6% to 29.3%. Men who were more controlling were more likely to abuse their female partners. The results reveal that partner violence against women is a significant public health issue in Thai society that must be addressed.

Violence against women is a significant problem and a universal phenomenon around the world, including in Thailand. One in 3 (35%) women around the world have experienced physical and/or sexual violence by their partner in their lifetime (WHO 2016). The United Nations defines violence against women as any acts of violent behavior that results in physical, sexual or psychological harm to women (UN 1993). There are many forms of violence against women. One of the most common forms is Intimate Partner Violence (IPV) (WHO 2002). IPV is a major public health issue, a hidden social problem, and women’s human rights violation (WHO 2016), (WHO 2005a) which negatively affects women’s physical, mental, sexual and/or reproductive health (WHO 2016).

## Intimate Partner Violence in Thailand

In Thai society, IPV is considered as a private issue and family matter because the family is an important social institution, and portrayed as a space of love and care, however, in reality family violence exists. Most Thais learn they should not tell outsiders about internal family matters. As a result, IPV remains an invisible and unrecognized issue in Thai society and women victims have to deal with their intimate violence in their lives alone (Archavanitkul et al. 2005). When violence occurs in families, it is not reported, thus the statistics on IPV in Thailand are likely to be underestimated. Most of IPV statistics in Thailand are reports from Government Organizations (GOs) and Non-Government Organizations (NGOs) such as the One Stop Crisis Centre (OSCC). The OSCC is run by the Ministry of Public Health and is a unit in government hospitals aimed to assist victims of violent situations from police station, NGOs’ and shelters. Individuals who notify the police or visit a hospital are likely severe cases of violence. Statistics show that the numbers of domestic violence cases have significantly increased from 25,767 to 31,866 between 2010 and 2013 respectively (Thai Health Promotion Foundation 2011), (The Women's Affairs and Family Development 2013). In addition, the report from the OSCC, Ministry of Public Health (MOPH) found that, in 2015, there were 23,977 women who used their services. Furthermore, there were 460 cases of partner violence wherein police were notified and 373 cases which were sent to the court (The Women's Affairs and Family Development 2015).

To address intimate partner violence, in 2007, Thailand launched “Domestic Violence Victim Protection Act, B.E. 2550” and amended the criminal law B.E. 2550 section 276 (Penal Code Amendment Act (No.19) B.E. 2550 (2007) 2007). The “Domestic Violence Victim Protection Act, B.E. 2550” comprises 18 sections; many sections are important to victims, perpetrators, and government officers. For example, section 4 states that “Whoever conducts any act of domestic violence or is said to commit domestic violence shall be liable to imprison for a term of not exceeding six months or to a fine of not exceeding six thousand Baht or both”; section 5 stats that “A domestic violence victim or a person who has found or known of domestic violence shall have the duty to notify a competent official for the execution of this Act..” Prior to 2007, the criminal law B.E. 2550 section 276, did not include marital rape as a crime so spouses were not legally protected against sexual abuse by their partner (Penal Code Amendment Act (No.16) B.E. 2546 (2003) 2003). However, changes to the law in 2007 added legal protection for spouses who are victims of sexually violence by their partner.

### I**ntimate Partner Violence Research in Thailand**

The WHO Multi-country study on Women’s Health and Domestic Violence Against Women (2005) examined the prevalence of intimate partner violence in 10 countries: Bangladesh, Brazil, Ethiopia, Japan, Namibia, Peru, Samoa, Serbia, Tanzania, and Thailand.

The study found that of the 24,097 women participants, 16.0% to 61.0% had experienced some act of physical partner violence in their lifetime. Sexual violence was reported by 6.0% to 58.0% of respondents. In addition, 16.0% to 69.0% of women reported that they had experienced either sexual and/or physical violence by their partners. In Thailand, the study found that 22.9% of women in urban areas reported physical violence, 29.9% reported sexual violence, and 41.1% reported physical or sexual violence, or both. Whereas, 33.8% of women in rural areas reported physical violence, 28.9% reported sexual violence, and 47.4% reported physical or sexual violence, or both. It is interesting to note that urban Thai women reported more sexual violence than physical violence. The percentage of both urban and rural women revealing one or more acts of controlling behaviors by male partner varied from 4.7% to 31.3%. Furthermore, in all settings, women who had experienced either physical and/or sexual violence regularly reported more emotional suffering and identified physical health issues such as pain (WHO 2005b).

In 2006, there was a cohort study on domestic violence among pregnant Thai women in one province. They recruited 421 women in third trimester of pregnancy and followed them until 6 weeks postpartum. The study revealed that 53.7% reported psychological violence, 26.6% faced physical violence, and 19.2% confronted sexual violence by their partner during the current pregnancy. Whereas, in the postpartum period respondents who had experienced some type of intimate partner violence ranged from 9.5% to 35.4% (Sricamsuk 2006).

The latest National Reproductive Health Survey from 2009 (Social Statistics Bureau 2010) revealed that the rate of intimate partner violence among Thai women across the country was 2.9%. More recently, Chuemchit and Perngparn (2014) reported that between July – December, 2010 there were 471 women in Bangkok city who used the services at One Stop Crisis Centre and more than 70% of women had been victimized more than once (Chuemchit and Perngparn 2014).

These data are the tip of the iceberg; the true extent of the issue cannot be seen. In Thailand, there have been no large-scale IPV prevalence studies since the 2005 WHO Multi-country study on Women’s Health and Domestic Violence Against Women. In response, the aim of this study was to examine the current prevalence of the various forms of intimate partner violence, including physical, psychological, sexual violence, and controlling behaviors and to identify factors associated with partner violence.

## Method

### Participants

This study was a cross-sectional study in 4 regions of Thailand: central, northern, southern, and northeastern which are the official government-categorized zones. A multi-stage sampling technique was used; first, simple random sampling was used for selection of one province in each region, second, all districts from the region were chosen for the study.

The sample size was calculated by n_*stra*_ *=* $\frac{N\sum {N}_h{\pi}_h\left(1-{\pi}_h\right)}{N^2{D}^2+\sum {N}_h{\pi}_h\left(1-{\pi}_h\right)}$, π = The proportion of IPV in Thailand (5.13%), based on National Statistic Office 2006. Therefore, a total sample size of 2,462 eligible persons was sought. Then the sample was calculated as proportionate to the size of each province and district. Finally, convenience sampling was used to select the participants at each community site. At the community level, first, researchers went to see a community leader to request the names and addresses of married or cohabiting women. Second, researchers visited women’s houses based on the list to conduct interviews. All eligible women we met were select as respondents. While both men and women can be victims, women are more likely than men to face various forms of partner violence and report IPV-related injury (Hegarty 2000), (Breiding et al. 2008), (Tjaden and Thoennes 2000). Therefore, this study focused on women age 20–59 years, still married or cohabiting with a partner, and willing to participate in the study. Table 1 provides a brief description of the study areas.

**Table 1:** Study sites by region and province*

Region	Female population (20–59 years old)	Province	Sample size
Central	5,113,691	Chonburi: Famous province for Oceanside. Lots of factory and tourism business. 80 km from Bangkok	725
Northern	3,644,532	Phitsanulok: 383 km from Bangkok. Mountainous area mostly in Agriculture	457
Southern	2,659,222	Surat Thani: Large City in the region. Center for tourism along seaside. Mostly in tourism and fishery business. 639 km from Bangkok	378
Northeastern	6,595,363	Khonkaen: Big city and the center of northeastern. Mostly in agriculture and tourism service business. 449 km from Bangkok	902
		Total	2462

*Thailand consist of 77 provinces

### Procedure and Data Collection

Each province had research assistants under the supervision of the principal investigator. In order to ensure quality data was captured, the female research assistants recruited for the data collection had at a minimum a bachelor degree, worked in the area of public health, and were experienced in community- based research. In each site, the interviews were conducted by trained interviewers who passed the standardized training based on WHO women’s health and domestic violence study (Jansen et al. 2004) covering issues of gender-based violence and its consequences, gender sensitivity, interviewing techniques and skills, ethic, and the questionnaire. To ensure the confidentiality and safety of participants, each participant had a separate and private interview by a trained female interviewer. All these processes were intended to protect the privacy of the participants and minimize the shame of respondents for disclosing details of their relationship. After finishing the interview, all respondents received useful information about available services, for instance, hotline call center, shelter houses, and OSCCs in each province. Interviewers also provided respondents with additional support from local health service providers if requested. The data collection was conducted in 2016.

Ethical permission for this study was obtained from the Ethics Review Committee for Research Involving Human Research Subjects, Health Science Group, Chulalongkorn University (COA No.201/2016), Thailand.

### Measurement Tool

A questionnaire was developed from the WHO multi country study on women’s health and domestic violence (Archavanitkul et al. 2005), (Garcia-Moreno et al. 2006). The WHO multi country study on women’s health and domestic violence, measures three items of “sexual abuse”, six items of “physical abuse”, four items of “psychological abuse”, and seven items of “controlling behaviors” which were examined separately from psychological abuse. After the pre-test, the Cronbach alphas for this measurement tool was 0.94.

The participants were asked questions related to their experience of specific acts of psychological, physical, and sexual abuse by their current husband and/or cohabiting male partner. Furthermore, we also followed the WHO framing of the questions which highlighted “how partner’s treat each other rather than so-called conflict negotiation” (Garcia-Moreno et al. 2006), (Heise and Garcia-Moreno 2002) because much intimate abuse in the Thai context can be conceptualized as punishment.

The questions on psychologically violent acts focused on insulting, humiliating, scaring, and threatening behaviors. The questions on physically violent acts were categorized as: 1) mild-to-moderate violence and 2) severe violence based on physical injury. Mild-to-moderate violence included pushing, shoving, grabbing or slapping and severe violence included choking, kicking, or using a weapon (WHO 2005b) (see Table 2). Sexually violent acts included using physical force for sexual intercourse, having sexual intercourse against women’s will, and sexual humiliation. For each act of violence, each participant was asked whether it had occurred over a year ago or within the year and then asked about the frequency of each act: 1) once or twice 2) a few times or 3) more than five times. The lifetime prevalence of IPV was defined as women who reported any kind of violent experience by a current partner over a year before the interview. Current prevalence was defined as women revealing at least one act of partner violence during the past year.

**Table 2:** Items of the questionnaire used to define psychological, physical, sexual violence, and controlling behaviors by intimate male partner

Psychological violence	Physical violence	Sexual violence	Controlling behaviors
Insulted or made feel bad about oneself	Mild-to-moderate:	Physically forced you to have sex when you did not want	Tried to keep you from seeing friends
Humiliated or belittled in front of other people	Slapped or threw something that could hurt	Had sexual intercourse when did not want because afraid of what partner might do	Tried to restrict you to contact your family
Did things to scare or frighten	Pushed or shoved	Was forced to do sexual activity that degrading or humiliating	Insisted on knowing where you were at all times
Threatened to hurt you or someone you care about	Severe violence:		Ignored you and treated you indifferently
	Hit with fist or something that could hurt		Got angry if you spoke with another man
	kicked, dragged, or beaten up		Was often suspicious that you were unfaithful
	Chocked or burnt		Expected you to ask permission before going out
	Threatened to use weapon		

In addition, this study also focused on seven forms of controlling behaviors by women’s male partners which consisted of various acts to force and restrict women’s daily life. For each act of controlling behavior, each respondent was asked whether it had happened within the past year (Yes or No). “Yes” responses were given a score of one and “No” responses were given a score of zero. Total scores were categorized into four levels (Garcia-Moreno et al. 2006): (1) None (2) low (3) medium and (4) high.

#### Data Analysis

All data was entered, cleaned and coded before analyzing. Statistical Package for Social Sciences (SPSS Version 22) was used to perform Uni-variate, Bivariate, and multivariate analysis. Uni-variate analysis was used to describe and summarize variables and find patterns in the data. (e.g. frequencies, percentages, means and standard deviation). Bivariate and multivariate analysis were used to test relationships between variables. For bivariate analysis, Pearson’s Chi square test with statistical level of *P* value <0.05 was used to analyze the association between controlling behaviors by male partner and experiences of violence. Multivariate regression was used to examine differences in lifetime IPV by demographic variables.

## Results

In this study, four provinces from 4 regions were sampled according to the proportion of the population. The mean age of the respondents was 39.4. Thirty-four percent of women had finished higher education, the rest had completed high-school, primary school, secondary school, and no education, respectively. About 10.7% of participants were housewives and/or no occupation, while 89.3% had an occupation (See Table 3).

**Table 3:** Selected socio-demographic characteristics of 2462 married/cohabiting women of the 4 regions of Thailand

Characteristics	n	(%)
Province (*n* = 2462)		
Chonburi	725	(29.4)
Phitsanulok	457	(18.6)
Surat Thani tourism	378	(15.4)
Khonkaen	902	(36.6)
Age (*n* = 2441)		
20–29 years	487	(20.0)
30–39 years	712	(29.2)
40–49 years	766	(31.4)
50–59 years	476	(19.5)
Mean ± SD	39.4 ± 10.2	
Range	20–59 years	
Education (*n* = 2396)		
No education	25	(1.0)
Primary	576	(23.5)
Secondary	398	(16.3)
High school	608	(24.8)
Higher education	840	(34.3)
Occupation (*n* = 2458)		
Housewife/ no occupation	262	(10.7)
Agriculture	521	(21.2)
Permanent employee	682	(27.7)
Temporary employee	144	(5.9)
Business owner	242	(9.8)
Company staff	221	(9.0)
Government officer	338	(13.8)
Other	48	(1.9)

Results demonstrate that out of 2,462 respondents, 15.4% had encountered psychological, physical, or sexual violence, suggesting that 1 in 6 married or cohabiting Thai women have experiences intimate partner violence in their lifetime (See Table 4).

**Table 4:** The proportion of married/cohabiting women who revealed facing violent experiences by male partner

Experiences of violence	n	(%)
Never	2,083	(84.6)
Ever	379	(15.4)

Table 5 reflects the proportion of married/cohabiting women who disclosed having experienced psychological, physical, or sexual intimate partner violence in their lifetime (over 1 year ago) or currently (within the past year). The study revealed that the most commonly reported type of partner violence was psychological violence. Sexually violence by a partner was noticeably less prevalent than the other 2 types of violence. Mild to moderate physical violence were more prevalent than severe physical violence.

**Table 5:** Lifetime (over 1 year ago) and current prevalence (with past year) of psychological, physical, or sexual intimate partner violence among married/cohabiting Thai women

Violent Act	Abused women (%)	Over 1 year ago (%)	Past year (%)
Psychological			
Insulted or made feel bad	(14.8)	(39.5)	(60.5)
Humiliated or belittled	(10.9)	(39.6)	(60.4)
Did things to scare	(15.4)	(38.1)	(61.9)
Threatened to hurt	(7.5)	(31.6)	(68.4)
Physical			
slapped or threw	(8.3)	(40.6)	(59.4)
Pushed or shoved	(10.6)	(47.5)	(52.5)
hit with fist	(5.4)	(46.9)	(53.1)
kicked, dragged, beaten up	(4.1)	(40.4)	(59.6)
Chocked or burnt	(2.6)	(34.9)	(65.1)
Threatened to use weapon	(4.4)	(43.4)	(56.6)
sexual			
physically forced you to have sex	(5.4)	(37.4)	(62.6)
Had sexual intercourse because afraid of	(10.4)	(37.3)	(62.7)
was forced to do sexual activity that degrading or humiliating	(3.3)	(36.2)	(63.8)

The most common form of psychological violence was being scared (15.4%), followed by being insulted (14.8%), being humiliated or belittled (10.9%), and being threatened (7.5%). Reports of physical violence included being pushed or shoved (10.6%), follow by being slapped or thrown (8.3%), being hit with a fist (5.4%), being threatened with a weapon (4.4%), being kicked/dragged/beaten up (4.1%), being chocked/burnt (2.6%). Reports of sexual violence, found that 10.4% of respondents revealed unwanted sexual intercourse, 5.4% were physically forced to have sex and 3.3% were forced to do sexual activities that were degrading or humiliating.

There were considerable differences between the proportion of women who experienced lifetime violence and current violence. More than 60% of respondents faced all forms of psychological violence and all forms of sexual violence in the past year, more than half (>50%) had experienced both mild to moderate and severe physical violence in the past year as well.

The lifetime prevalence of IPV varied by the type of violence experienced. Psychological violence varied from 31.6% (threatened to hurt you or someone you care about) to 39.6% (humiliated or belittled in front of other people); physical violence varied from 34.9% (chocked or burnt) to 47.5% (pushed or shoved); whereas, sexual violence varied from 36.2% (was forced to do sexual activity that was degrading or humiliating) to 37.4% (physically forced you to have sex when you did not want). Most respondents reported repeated acts of IPV.

Tables 6 and 7 show the proportion of married/cohabiting women who reported having experienced controlling behaviours by an intimate partner and highlight the association between experiences of violence and controlling behaviours. The most frequently reported act of controlling behavior by a male partner was “insisted on knowing where the female partner was at all times” (29.3%) followed by “got angry if female partner spoke with another man” (28.5%), “suspicious that female partner is unfaithful” (21.3%), “ignored and treated indifferently” (16.5%), “keeps female partner from seeing friends” (15.2%), “expected female partner to ask permission before going out” (10.7%), and “tried to restrict female partner to contact your family” (4.6%).

**Table 6:** Portrays the proportion of married/cohabiting women who reported having experienced controlling behaviours by an intimate partner

Controlling behaviours	n	Ever		Never	
		n	%	n	%
Tried to keep you from seeing friends	2461	374	15.2	2087	84.8
Tried to restrict you to contact your family	2460	114	4.6	2346	95.4
Insisted on knowing where you were at all times	2462	722	29.3	1740	70.7
Ignored you and treated you indifferently	2461	405	16.5	2056	83.5
Got angry if you spoke with another man	2462	702	28.5	1760	71.5
Was often suspicious that you were unfaithful	2462	524	21.3	1938	78.7
Expected you to ask permission before going out	2462	264	10.7	2198	89.3

**Table 7:** Controlling behaviours by intimate male partner reported by married/cohabiting women

Number of married/cohabiting women	Experiences of violence	Act of controlling behaviours				P*	Number of Act, mean	P^⍶^
		None (%)	1 (%)	2 or 3 (%)	4–7 (%)			
2,079	Never	56.2	17.7	17.9	8.2		1.0	
379	Ever	21.6	13.5	25.6	39.3	<0·0001	2.6	<0·0001

The percentage presents controlling behaviours act regard to partner violence experience. *Pearson χ2 2 × 4 table. ⍶ ANOVA

The percentage of women who reported one or more acts of controlling behaviors by their male partner varied from 13.5% to 39.3%, which suggests that the level of male control over female behavior is normative to a certain degrees. The respondents who had experienced IPV were significantly more likely to have also experienced controlling behavior by their male partner than women who had not faced partner violence.

Table 8 illustrates multivariate logistic regression models to determine relationships between lifetime IPV victimization and demographic variables. There were significant differences in IPV prevalence among provincial settings. Compared to women in Chonburi province (central), Phitsanulok province (northern) were significantly more likely to have experienced lifetime IPV victimization (OR 2.34; 95% CI = 1.34–4.06). Women who worked in “white collar” occupations were significantly less likely to report lifetime IPV victimization than women who worked in “blue collar” occupations (OR 0.66; 95% CI = 0.52–0.85). Women who completed higher education were significantly less likely to report lifetime IPV victimization than those who had no education and those who completed only primary education (OR 0.37; 95% CI = 0.15–0.91) and (OR 0.66; 95% CI = 0.49–0.89), respectively). Women who had sufficient income and savings were significantly less likely to report lifetime IPV victimization than those who had insufficient income and those who had sufficient but no saving (OR 0.26; 95% CI = 0.17–0.41) and (OR 0.46; 95% CI = 0.30–0.72), respectively).

**Table 8:** Relationship between lifetime IPV victimization and demographic variables

	Lifetime IPV	
	OR	95% CI
Province		
Phitsanulok	2.335	1.34–4.06
Khonkaen	0.36	0.26–0.49
Suratthani	0.19	0.13–0.27
Chonburi	Ref	Ref
Age (years)		
20–29	1.29	0.92–1.81
30–39	1.31	0.96–1.79
40–49	1.29	0.95–1.76
50–59	Ref	Ref
Occupation		
Housewife	1.78	0.52–1.15
Blue collar	0.66	0.52–0.85
White collar	Ref	Ref
Education		
None	0.37	0.15–0.91
Primary school	0.66	0.49–0.89
High school	0.79	0.60–1.03
Higher education	Ref	Ref
Years of conjugal living		
< 10	1.10	0.73–1.66
11–20	1.12	0.72–1.73
21–30	0.95	0.61–1.47
> 30	Ref	Ref
Income		
Insufficient	0.26	0.17–0.41
Sufficient but no saving	0.46	0.30–0.72
Sufficient and saving	Ref	Ref

## Discussion

According to this study 15.4% of married/cohabiting Thai women have experienced psychological, physical, and/or sexual intimate partner violence at some point in their lives. This number is considerably higher than the latest National Reproductive Health Survey in 2009 (Social Statistics Bureau 2010), which found that the national prevalence of partner violence among Thai women was 2.9%. This wide discrepancy in results could be due to the measurement tool. The 2009 survey asked only one question about experience of physical partner violence in the past 12 months, if the response was yes, then there were 2 following questions: 1) what was the reason for the violence and; 2) have you ever asked for any help. This single question is a limitation of the previous survey because intimate partner violence can be psychological, physical, and/or sexual violence and surveys should ask behaviorally specific questions in order to encourage respondents to disclose the entire scope of their violent experiences (Garcia-Moreno et al. 2006), (Straus et al. 1996), (Ellsberg et al. 2001).

This study also showed that across the country, psychological violence had the highest prevalence followed by physical violence and then sexual violence. Similar findings have been recorded in the WHO multi-country study (Garcia-Moreno et al. 2006) on women’s health and domestic violence, across 10 countries, sexual violence was greatly less prevalent than physical violence. However, Thailand was an exception with rates of sexual violence higher than physical violence in the WHO multi-country study. Forty-four percent of women in the city and 29% of women in the province had experienced sexual violence by their partner (Archavanitkul et al. 2005), (Garcia-Moreno et al. 2006), (Heise and Garcia-Moreno 2002).

To explain these contradictory results, we had look back over the past decades, particularly the Thai policies and campaigns on IPV prevention. For example, “Domestic Violence Victim Protection Act, B.E. 2550” and “Amended the criminal law B.E.2550 section 276”. Prior to the amended law, section 276 stated that “any person who commits sexual intercourse with a woman who is not his wife, and against the latter’s will, by threatening her, or doing any act of violence…, shall be punished with imprisonment…” (Penal Code Amendment Act (No.16) B.E. 2546 (2003) 2003). However, changes to the law, by removing the sentence “with a woman who is not his wife”, added protection to spouses against sexually violence by their partners (Penal Code Amendment Act (No.19) B.E. 2550 (2007*)* 2007). These tools may gradually reduce the prevalence of sexual violence in Thai society but do not appear to have had a major impact over the last 10 years. However, today the campaigns related to IPV prevention are rarely seen in mainstream Thai society.

All of the women who reported physical violence had experienced more violence in the past year than over her lifetime. These study results confirm that IPV is a common experience worldwide. In a Japanese city, the lifetime prevalence of physical abused by an intimate partner was 13%, whereas, in Bangladeshi city it was 40% (Garcia-Moreno et al. 2006). In Vietnam, the prevalence rate of physical partner violence was 30.9% (Vung et al. 2008). In Cambodia, 25% of women interviewed experienced partner violence since marriage, with 23% disclosing such violence in the prior year (Yount and Carrera 2006). Whereas, in Canada 6% of women revealed having been abused by their male partner (McCormick et al. 2016). In the USA, 5.3% of postpartum women stated that they were physically victimized by an intimate partner in the year prior to becoming pregnant (Stewart et al. 2017). A WHO Study recorded that the 12-month prevalence of partner violence was much higher in developing countries as compared to developed countries such as Canada and the USA (McCormick et al. 2016), (Stewart et al. 2017) which suggests that a good legal framework, effective government campaigns, sufficient shelters and women’s individual empowerment are important and provide a foundation from which women have more resources to choose to withdraw from abusive relationships. Findings that emphasize the importance of resources and social status have been noted in Vietnam (Yount and Carrera 2006) and others, and suggest that partner violence might be different in settings of low income and unemployment status compare with higher socio-economic settings (Mallory et al. 2016). Finally, controlling behaviors by male partners were significantly associated with partner violence, which is consistent with WHO findings showing that men who are a perpetrators also show higher rates of controlling behaviors than men who do not abuse their partner (Garcia-Moreno et al. 2006). Therefore, resources to support women coupled with campaigns to educate men and deemphasize traditional gender norms may be critical to reducing rates of IPV in Thailand.

### Limitations

This study only asked about rates of IPV against women, although women may also be perpetrators of violence in relationships. However, research has consistently demonstrated that men are the dominant perpetrators of violence in relationships including married and cohabiting relationships (Archavanitkul et al. 2005), (Garcia-Moreno et al. 2006), (Zhang et al. 2012). Some studies have documented that women are almost eight times more likely to be victims of violence by a partner than men (Stuckless et al. 2015). Another limitation was this study based on self-report, which may be affected by recall bias, as well as cultural biases in disclosure which affect to participants’ willingness to reveal their violent experiences. We recognize that in the Thai context, IPV is viewed as a family affair. The privacy of the family in Thai society strengths gender power imbalance and hierarchies that are often dependent on the sustainability of male domination and violence (Mohamad and Wieringa 2014). This research highlights the prevalence of psychological, physical and sexual intimate partner violence in Thai society. Although some differences in prevalence were noted in the results according to education, occupation, and number of partners these differences did not account for the varying rates of violence across provinces.

### Strengths

This study had significant methodological strengths including: using a standardized questionnaire, pre-testing the questionnaire, training interviewers, and collaborating with communities to account for the ethical complexities of working on sensitive issues within communities in Thailand. All these methodological techniques may help to reduce bias in the study and increase disclosure. However, we believe that in Thai culture, violence is a deeply personal issue which likely leads to an underestimation of the overall violence situation in Thai society. Hence, the findings of this study should be thought of as an underestimation of the actual rate of intimate partner violence in Thailand.

### Recommendations

This study confirms the high prevalence of intimate partner violence in Thailand which is a serious human rights violation and highly significant public health issue as IPV is linked to many negative health consequences. These results should encourage national action to prevent IPV and contribute national data to a spectrum of interest groups including policy makers, public health practitioners, multi-disciplinary, educators, media, activists, and communities. National policy change should work to eliminate the root causes of violence against women and re-build Thai society as violence free society. For instance, the government needs to prioritize the establishment of a special agency solely responsible for providing a comprehensive package of services to women suffering from various forms of violence. The services must include: counseling, shelter, rehabilitation and referrals to additional health and social support services and professional sensitivity training for multi-disciplinary practitioners on issues concerning violence against women and the enhancement of a public campaign through the mass media and social media to eradicate all forms of violence against women. Moreover, we must challenge social norms to ensure that domestic violence is a public rather than a private issue and create networks and community participation to prevent and to monitor domestic violence in the community. Media messaging should focus on promoting gender equity, respect for human dignity and non-violent relationships. In particular, media should work to avoid reproducing structural and cultural violence against women in media reporting and avoid producing content that reinforces and motivates violence.
